# Attitudes and practices toward feral cats of male and female dog or cat owners and non-owners in Seoul, South Korea

**DOI:** 10.3389/fvets.2023.1230067

**Published:** 2023-10-24

**Authors:** Sun-A. Kim, Claire J. Kenyon, Sejin Cheong, Jenna Lee, Lynette A. Hart

**Affiliations:** ^1^Clinical Animal Behavior Service, Veterinary Medical Teaching Hospital, Chungbuk National University, Cheongju, Republic of Korea; ^2^School of Veterinary Medicine, University of California, Davis, Davis, CA, United States; ^3^Department of Population Health and Reproduction, School of Veterinary Medicine, University of California, Davis, Davis, CA, United States

**Keywords:** neutering, human-animal interactions, Trap-Neuter-Return, felines, canines

## Abstract

The number of pet cats in South Korea has sharply increased since 2010. Problems have arisen with feral or stray cats, creating conflict among residents, to such an extent that the government provides some sites for people to offer feeding stations for the stray cats. This study investigated hypotheses on people’s attitudes toward feral cats in Seoul, South Korea: (i) dog and cat owners would show more positive attitudes than non-owners toward feral cats; (ii) females would have more positive attitudes toward feral cats than males; (iii) the number of Seoul-provided feeding stations by district would be correlated with people’s positive attitudes toward feral cats. Responses from 7,394 participants were used for the final analyses with 3,179 males, 3,607 females, and 599 others (includes “decline to state”). Cat owners reported more extremely positive attitudes toward feral cats than people who had no cats. Females more often had cats than males, and they were more extremely positive toward pet and feral cats than males, and strongly opposed to culling as a management strategy. The attitudes toward feral cats of people with only dogs were intermediate between people with cats and people without pets, more resembling those of people without pets. There was a correlation between the number of city-provided feeding stations and people’s attitudes toward feral cats, but only in the areas with at least 40 feeder stations; having many city-provided feeding stations was associated with very negative attitudes to feral cats. Whether the very large number of feeding stations were provided in the two neighborhoods due to a previously excessive number of feral cats in those neighborhoods, vs. those feeding stations attracting or provisioning an ever-growing number of feral cats, is unknown. These results show sharp differences in attitudes between cat owners and non-owners, and between males and females. Results resemble findings in other studies, perhaps with more extreme differences between sub-groups. The study revealed that attitudes toward pet and feral cats in Seoul were complex and varied with pet ownership, with sex, and with neighborhood context.

## Introduction

Genetic and archeological evidence suggests that the domestication of cats began as early as 10,000 years ago when agriculture was advancing (1). Throughout their long history with cats, people have held attitudes and beliefs toward cats that differ significantly across cultures and religions (2). In Egypt, cats were considered sacred and were worshiped as deities (2), whereas some other cultures and religions considered cats to be associated with bad luck and bad spirits, as in old folklore of Korea (3). Both extremely positive and negative feelings about cats are reported in Brazil (2). In modern society, regardless of culture or religion, people’s attitudes toward cats vary drastically, from those who love cats to those who despise them. These conflicting attitudes play out differently in various locations. Increasingly, with the emphasis on no-kill and the intrinsic value of animals (4), eradication may be viewed as an infeasible policy, even on small islands (5); Trap-Neuter-Return (TNR) and similar approaches often become primary strategies. At the same time, wildlife professionals highlight that cats are an invasive species and recommend preventing outdoor feeding of cats and not allowing them to roam freely (6). Serious efforts have been made to bridge the values conflicts, with wildlife advocates perceiving stray cats as an invasive species and cat advocates viewing them as homeless pets. A 2020 paper by Leong et al. provided explanatory diagrams clarifying the complexity of these issues and conflicts (7). One diagram depicts how human sources of outdoor cats are enhanced by human provisioning of the cats, leading to a general outdoor cat problem, then resulting in many specific problems that groups want to address. A second diagram shows the many different measures required to mitigate the problems, starting with keeping pet cats indoors and stopping abandonment.

Problems with cats have accelerated in South Korea with the recent surge in cat ownership. An estimated 1.54 million households among a total of 21.5–23.4 million households were reported to have 2.6 million cats in 2020: a noticeable increase from the 0.6 million cats in 2010 with 17.5 million households, and 1.9 million reported in 2015 with 19.6 million households (8–11). Despite the growing popularity of pet cats, stray or feral cats have become a focus of national conflict in South Korea (12). Stray cats are defined as pets that were once raised and socialized by humans. Feral cats, in contrast, have had little to no human contact in their lives and are essentially wild. In an urban environment, it is difficult to differentiate stray cats from feral cats, thus, the term feral cats will be used in this paper.

Common complaints against feral cats in South Korea resemble those in other countries such as the United States or Japan, where conflicts focus on cats’ excrement deposition, scavenging for food in the trash, and cries made during territorial disputes among feral cats and during mating (13, 14). These complaints are especially impactful in urban settings. Studies in Guelph, Canada, clarified how complicated the issues are, especially with urban feral cat colonies (15). Cat owners often are reported as more favorable to feral cats than non-owners, as with an example in California (16). The research in Canada reported that cat owners had more favorable attitudes to feral cats than non-owners; non-owners favored euthanasia of the feral cats more often than owners (17). This group recommended using community-wide approaches (18).

Additionally, females have been reported as more sympathetic to feral cats than males and less willing to consider lethal options when dealing with feral cats. In a study in Bulgaria, 33% of females fed stray cats, and only 20% of males (19). A study in Australia found that males were more willing than females to use all control methods, including poison and methods that may be inhumane; females also were more reluctant to use methods that were unfamiliar or unknown to them (20). Another study in Australia also reported that males were more accepting of lethal methods than females (21). A large study in Belgium found numerous differences in attitudes of males and females toward managing cats, leading the authors to conclude that customized approaches were needed for varied sub-groups (22).

Opinions differ on how best to address problems with feral cats. Studies in Hawaii by Lohr and colleagues have found high acceptance of lethal traps as the best technique for dealing with feral cats, and TNR as the worst technique, reflecting the costs and benefits; however, respondents felt that avoiding abandonment of cats would be even better (23). These results differ from a general preference for non-lethal methods of dealing with feral cats (4). While recreating their study in Australia, Lohr’s group found it challenging to monitor feral cats at a vast landscape scale (24). Several recent studies describe the advantages of neutering feral cats. One highlights that the smaller territories of cats in protected island settings are better for wildlife (25). Others report that smaller territories result in less aggression (26) and result in fewer injuries for males (27). Those in South Korea who despise feral cats have demanded that feeding and caring for the cats be stopped and some have advocated for the culling of feral cats. Some have even resorted to violence and hate crimes against both advocates and animals.

Some committed animal advocates work for the welfare of feral cats, such as by providing feeding stations, food, shelter, and medical help to feral cats. Such animal advocates are sometimes described as “semi-owners” (28). Feeding stations are designated patches of space around neighborhoods where dedicated volunteers create and supply a small shelter with food and water. These volunteers also keep tabs on the well-being of cats and provide medical attention and TNR services to the cats in need. Previous studies have also found that providing and managing feeding stations for feral cats makes it easier to estimate the population size, identify immigrant cats, provide medical attention to feral cats, and conserve wildlife (29, 30). Helback’s study demonstrated feral cat population densities correlated with providing feeding stations, so potentially feral cat habitats can be maintained and the cat populations successfully managed in designated areas (30).

After considering the advantages of feeding stations and to alleviate human conflicts caused by feral cats, the Seoul Metropolitan City Government began employing cat feeding stations in 2013. This involves providing designated spaces on government property where people can provide food and water for cats. These stations facilitate the efforts of volunteer programs that aim to spay and neuter feral cats using the feeding stations. During this survey there were 346 feeding stations in 25 districts in Seoul. However, no reports have been presented on whether and how feeding stations relate to people’s attitudes toward feral cats.

The aim of this study was to investigate people’s attitudes toward feral cats in Seoul, South Korea, a city where petkeeping is not a longstanding tradition. We had three hypotheses: (i) people who had a pet would show more positive attitudes than non-owners toward feral cats; (ii) females would have more positive attitudes toward feral cats than males; (iii) there would be a correlation between the number of city-provided feeding stations and people’s positive attitudes toward feral cats. Considering the social conflict among residents in Seoul concerning feral cats, we sought to clarify the characteristics and attitudes of people supporting feral cats as compared with those opposed to feral cats.

## Methods

### Study design overview

The Institutional Review Board (IRB) of the University of California, Davis, ruled this study as exempt (IRB approval number: FWA No: 00004557). A web-based survey was conducted via an online survey site (Qualtrics), between August 2021 and January 2022 in South Korea; recruitments and responses were primarily from Seoul. It was distributed via social media (Facebook, YouTube, Instagram), in the Korean language. Responses were gathered from throughout South Korea and included in the general analyses. For the assessment of feeding stations in districts of Seoul, only responses from people residing in Seoul (69% of respondents) were included.

The survey consisted of a total of 24 questions, eight questions concerning general information on the respondents and their pet ownership history, eight questions regarding their attitudes toward feral cats, two questions for respondents’ preferences for the management of cats, and six questions about their experiences related to providing feeding stations and shelter. The full survey is available in Figshare. Questions for the survey were written in English by all authors, and the survey was translated into Korean by two veterinarians using a forward–backward translation procedure (31). The inclusion criteria for participants in the study included adult residents in Korea who are over the age of 18 years.

### Statistical analyses

A total of 24 items in the questionnaire included three binary (yes or no), seven categorical (e.g., gender, species of the participants’ first pet), 10 ordinal (e.g., attitudes toward pet cats or feral cats), three numerical (e.g., current age, age when the participant got their first pet), and one open-ended questions (i.e., describe any experiences related to animal shelters in Korea). Three categorical questions (i.e., options that the participants support for managing feral cat populations, species of their current pets, and characteristics that the participants find most important to be a good cat owner) allowed the participants to select all options that applied.

All the analyses were done in R (version 4.2.0). Descriptive statistics were used to summarize the survey results. Binary, categorical, or ordinal data were summarized as counts and percentages, and the percentages were calculated after excluding not-responded data for each question. For the question about gender, “Non-binary,” “Gender fluid,” “Other,” and “Prefer not to say” were re-categorized as a single item (“Others”); overall, fewer than 10% of respondents declined to provide a binary response. For ordinal data, “very negative,” “negative,” “neither positive nor negative,” “positive,” and “very positive” were converted into ordinal values “1–5,” respectively. Numerical data were summarized as means and standard deviations in each categorical group. To test for statistical differences in attitudes among four groups of animal owners (dog, cat, dog and cat owners, and no pet owners) and gender, the Kruskal-Wallis test was used with ordinal data. To examine the correlations between the respondents’ answers to attitudes toward pet cats and feral cats in the same owner or gender group, Spearman’s rank correlation coefficient was used. When results were significant (*p* < 0.05), pairwise comparisons were conducted using the Wilcoxon rank sum test. To assess attitudes toward feral and pet cats by the species of animal kept as a pet after adjusting for gender effect (male vs. female), logistic regression was used after re-categorizing the ordinal data (“very negative,” “negative,” “neither positive nor negative,” “positive,” and “very positive”) into binary data as “negative” including “very negative,” “negative,” and “neither positive nor negative” and “positive” including “positive,” and “very positive.”

## Results

The total number of respondents was 11,240. For inclusion, participants were required to answer over 95% of all the questions and to own only a cat and/or a dog, or not own any pet. Participants under 19 years old were excluded. Data for participants who reported a lower “current age” than “the age when they first got a cat” were excluded. Thus, reflecting these inclusion and exclusion criteria, the total number of participants whose data were used in the statistical analysis was 7,394. The general demographics of the participants and characteristics toward feral cats and management are summarized in Table 1.

**Table 1:** Characteristics of the participants grouped by species of their current pet (*n* = 7,394).

Characteristics count (percentage)		Cat (*n* = 2,831)	Cat and dog (*n* = 446)	Dog (*n* = 941)	No pet (*n* = 3,176)	Total (*n* = 7,394)
Gender	Male	567 (20.0%)	91 (20.4%)	489 (52.0%)	2,034 (64.0%)	3,179 (43.0%)
	Female	2,095 (74.0%)	332 (74.4%)	366 (38.9%)	814 (25.6%)	3,607 (48.8%)
	Others	164 (5.8%)	21 (4.7%)	85 (9.0%)	329 (10.4%)	599 (8.1%)
Age in years *Mean (SD)*		34.5 (10.1)	36.9 (11.7)	31.5 (9.6)	30.6 (8.5)	32.5 (9.7)
Age when owners got their first pet (including all species of animals) *Mean (SD)*		18.0 (11.4)	16.3 (10.4)	15.3 (9.0)	10.4 (7.1)	14.3 (10.0)
Owners’ attitudes toward intense feeding of cat colonies/management	Strongly disagree	643 (22.7)	101 (22.6)	549 (58.4)	2,185 (68.8)	3,478 (47.0)
	Disagree	118 (4.2)	22 (4.9)	83 (8.8)	415 (13.1)	638 (8.6)
	Neither agree nor disagree	154 (5.4)	24 (5.4)	30 (3.2)	165 (5.2)	373 (5.0)
	Agree	484 (17.1)	42 (9.4)	65 (6.9)	133 (4.2)	724 (9.8)
	Strongly agree	1,430 (50.5)	256 (57.4)	214 (22.8)	278 (8.7)	2,178 (29.5)
Whether owners were involved in the management of feral cats	Yes	1,000 (35.3)	212 (47.5)	131 (13.9)	184 (5.8)	1,527 (20.6)
	No	1,830 (64.6)	234 (52.4)	810 (86.1)	2,992 (94.2)	5,866 (79.3)
Three characteristics that owners find the most important to be a good cat owner*	Responsibility	2,481 (87.6)	410 (91.9)	820 (87.1)	2,767 (87.1)	6,478 (87.6)
	Knowledge about cat health	1,417 (50.1)	245 (54.9)	282 (30.0)	900 (28.3)	2,844 (38.5)
	Knowledge about cat behavior	1,321 (46.7)	238 (53.4)	316 (33.6)	964 (30.3)	2,839 (38.4)
Whether owners have seen cats in animal shelters in Korea	Yes	1,203 (42.5)	224 (50.2)	353 (37.5)	774 (24.4)	2,554 (34.5)
	No	1,627 (57.5)	222 (49.8)	587 (62.4)	2,401 (75.6)	4,837 (65.4)
Respondents living in certain districts of Seoul with greatest number responding**		Gangnam 149 (5.3) Gwanak 111 (3.9) Songpa 93 (3.3)	Gangnam 22 (4.9) Eunpyeong 14 (3.1) Songpa 14 (3.1)	Gangnam 46 (4.9) Gwanak 37 (3.9) Gangseo 37 (3.9)	Gangnam 161 (5.1) Gwanak 134 (4.2) Seongbuk 123 (3.9)	Gangnam 378 (5.1) Gwanak 295 (4.0) Seongbuk 227 (3.1)

*On this question, all items that applied could be selected. **31% of the respondents were living outside of Seoul and could not specify their regions. Nine of the 25 districts of Seoul had government-sponsored feeding stations. Data are shown for the three of these districts having the most respondents for each category of pet ownership.

**Figure 1:**
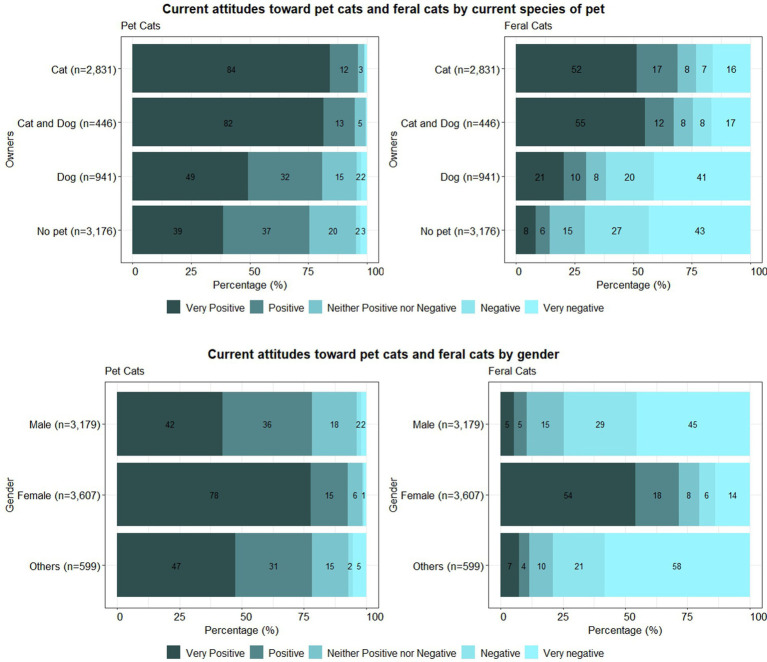
**(A)** Participants’ current attitudes toward pet cats and feral cats grouped by their current species of pets. **(B)** Participants’ current attitudes toward pet cats and feral cats grouped by gender.

Overall, as shown in Figure 1A, participants’ attitudes toward pet cats were more positive than toward feral cats, regardless of the species of animals they kept as pets. Cat owners were the most positive toward pet cats (84%, very positive), and cat and dog owners were the most positive toward feral cats (55%, very positive). However, 41% of dog owners and 43% of people who did not have pets evaluated their attitudes toward feral cats as “very negative.” As a result, for both pet cats and feral cats, significant differences among the owner groups were observed (*p* < 0.001). All the other pairwise comparisons were significant as well, except for the comparisons between cat owners and cat and dog owners in attitudes toward both pet cats (*p* = 0.16) and feral cats (*p* = 0.55); cat owners and cat and dog owners were similar in their attitudes toward pet cats and feral cats. Within each category group of ownership, the correlations between the answers to attitudes toward pet cats and feral cats were moderately correlated in owners of cat or cat and dog owners (dog: *r* = 0.36; cat: *r* = 0.41; cat and dog: *r* = 0.47; no pet: *r* = 0.22; *p* < 0.001).

Figure 1B shows the attitudes toward pet cats and feral cats depending on the participants’ gender; 54% of females answered “very positive” toward feral cats but less than 10% of males and the other group answered “very positive” toward feral cats. Interestingly, investigating the correlations between the answers to attitudes toward pet cats and feral cats within the same gender group, they were very weak in male (*r* = 0.18) and other (*r* = 0.18) groups, whereas the female group (*r* = 0.54) showed a moderate correlation, which means that the answers to the questions between pet cats and feral cats in male and other gender group were very inconsistent.

However, as in Table 1, the species of animal kept as pets were significantly associated with person’s gender group (*p* < 0.001); 52% of male participants were dog owners, and 74% of female participants were cat owners. Thus, Table 2 summarized the attitudes toward feral and pet cats by the species of animal within each female and male group. Male cat owners still had negative attitudes toward feral cats (negative – 20.3%, very negative – 31.7%), but their attitudes were less negative than those of male dog owners (negative – 31.3%, very negative – 53.6%), or non-owners (negative – 31.7%, very negative – 47.4%). In the final logistic regression model to assess attitudes toward feral and pet cats by the species of animal kept as a pet after adding gender effect (male, female), results showed that the odds of being positive toward feral cats were 93% lower in males than in females, although that of being positive toward pet cats was only 44% lower in the male than in the female group (Table 3). The odds of being positive toward feral or pet cats were significantly lower in dog owners or non-owners compared to the cat owner group, even after adjusting for the gender effect.

**Table 2:** Current attitudes toward feral cats and pet cats by person’s gender and species of the participants’ pets.

Current attitudes toward feral cats							
Females (*n* = 3,607)				Males (*n* = 3,179)			
Cat (2,095)	Very Positive Positive NeutralNegative Very Negative	1,324	63.2%	Cat (576)	Very Positive Positive NeutralNegative Very Negative	106	18.7%
		400	19.1%			74	13.1%
		124	5.9%			92	16.2%
		53	2.5%			115	20.3%
		194	9.3%			180	31.7%
Cat and dog (332)	Very Positive Positive Neutral Negative Very Negative	223	67.2%	Cat and dog (91)	Very Positive Positive Neutral Negative Very Negative	16	17.6%
		49	14.8%			5	5.5%
		21	6.3%			14	15.4%
		8	2.4%			21	23.1%
		31	9.3%			35	38.5%
Dog (366)	Very Positive Positive Neutral Negative Very Negative	176	48.1%	Dog (489)	Very Positive Positive Neutral Negative Very Negative	10	2.0%
		70	19.1%			18	3.7%
		25	6.8%			46	9.4%
		27	7.4%			153	31.3%
		68	18.6%			262	53.6%
No Pet (814)	Very Positive Positive Neutral Negative Very Negative	226	27.8%	No Pet (2,032)	Very Positive Positive Neutral Negative Very Negative	36	1.8%
		117	14.4%			67	3.3%
		124	15.2%			320	15.7%
		141	17.3%			645	31.7%
		206	25.3%			964	47.4%
Current attitudes toward pet cats							
Female (*n* = 3,607)				Male (*n* = 3,179)			
Cat (2,095)	Very Positive Positive Neutral Negative Very Negative	1,858	88.7%	Cat (576)	Very Positive Positive Neutral Negative Very Negative	413	72.8%
		184	8.8%			118	20.8%
		37	1.8%			30	5.3%
		1	<0.1%			3	0.5%
		15	0.7%			3	0.5%
Cat and dog (332)	Very Positive Positive Neutral Negative Very Negative	288	86.7%	Cat and dog (91)	Very Positive Positive Neutral Negative Very Negative	59	64.8%
		37	11.1%			21	23.1%
		7	2.1%			10	11.0%
		–	–			1	1.1%
		–	–			–	–
Dog (366)	Very Positive Positive Neutral Negative Very Negative	234	64.0%	Dog (489)	Very Positive Positive Neutral Negative Very Negative	193	39.5%
		78	21.3%			195	40.0%
		41	11.2%			80	16.4%
		8	2.2%			8	1.6%
		5	1.4%			13	2.7%
No Pet (814)	Very Positive Positive Neutral Negative Very Negative	426	52.3%	No Pet (2,032)	Very Positive Positive Neutral Negative Very Negative	681	33.5%
		239	29.4%			811	40.0%
		123	15.1%			448	22.0%
		9	1.1%			42	2.1%
		17	2.1%			50	2.5%

**Table 3:** Logistic regression results of the attitudes toward feral and pet cats associated with the person’s gender and species of animal kept as a pet.

Attitudes toward feral cats			
Variable	Odds Ratio	95% CI	*p* value
Gender			
Female	Ref		
Male	0.07	0.06 - 0.08	<0.001
Species of animal kept as pet			
Cat	Ref		
Cat and dog	0.87	0.64 - 1.13	0.3
Dog	0.31	0.25 - 0.37	<0.001
No Pet	0.14	0.12 - 0.17	<0.001
Attitudes toward pet cats			
Variable	Odds Ratio	95% CI	P value
Gender			
Female	Ref		
Male	0.56	0.48 - 0.67	<0.001
Species of animal kept as pet			
Cat	Ref		
Cat and dog	0.78	0.48 - 1.34	0.34
Dog	0.19	0.14 - 0.25	<0.001
No Pet	0.14	0.11 - 0.18	<0.001

**Figure 2:**
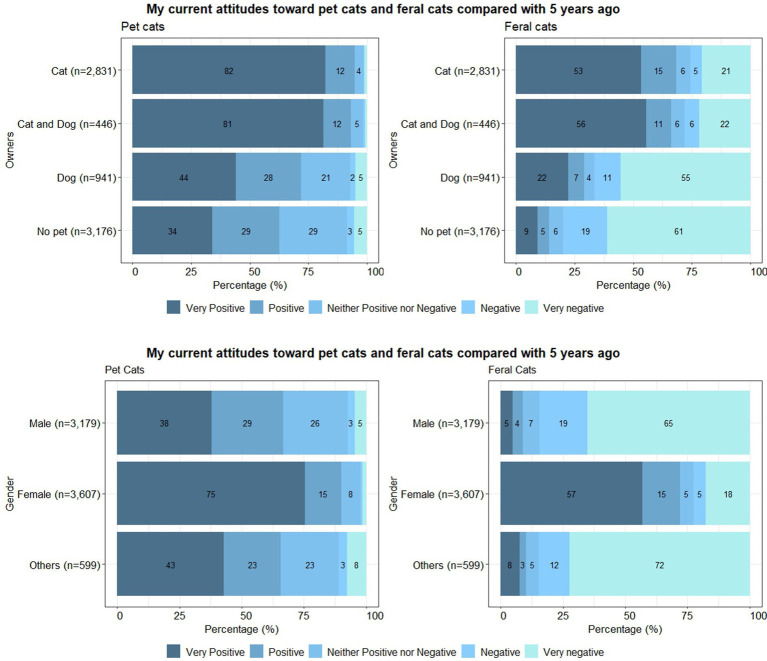
**(A)** Participants’ current attitudes toward pet cats and feral cats compared with 5 years ago grouped by their current species of pets. **(B)** Participants’ current attitudes toward pet cats and feral cats compared with 5 years ago grouped by gender.

Overall, as shown in Figure 2A, participants reported their current attitudes toward pet cats compared with 5 years ago as more positive than toward feral cats, regardless of the species of animals that they currently kept as pets. Especially, 55% of dog owners and 61% of people without pets evaluated their current attitudes toward feral cats compared with 5 years ago as “very negative”; compared to Figure 1A, the negative values were increased. Similarly, when seeing the answers by gender groups in Figure 2B, 65% of the male group and 72% of the other group evaluated their current attitudes toward feral cats compared with 5 years ago as “very negative,” reflected in increased values compared to Figure 1B. Current attitudes toward pet cats and feral cats compared with 5 years ago also showed significant differences among the owners and gender (*p* < 0.001). Also, all the other pairwise comparisons between owner groups were significant, except for the non-significant comparisons between cat owners and cat and dog owners for both their attitudes toward pet cats and feral cats; again, cat owners and cat and dog owners were similar in their attitudes. No significant differences in attitudes toward pet cats and feral cats between males and the other gender group were observed, but females showed significantly different attitudes compared to the two other gender groups (*p* < 0.001). The correlations between the answers to attitudes toward pet cats and feral cats within cat or cat and dog owners were moderately correlated (dog; *r* = 0.39, cat; *r* = 0.41, cat and dog; *r* = 0.45, no pet; *r* = 0.18; *p* < 0.001). As in Figure 1B, the males (*r* = 0.12) and the other (*r* = 0.11) gender group showed inconsistent weak associations between answers to pet cats and feral cats in Figure 2B. When comparing the attitudes toward feral and pet cats compared with 5 years ago by the species of animal within each female and male group, the results were similar to Table 2, but the answers were more polarized. Women had become more extremely positive, and men had become more extremely negative toward feral cats in all categories of owners, when they assessed themselves compared to 5 years ago.

**Figure 3:**
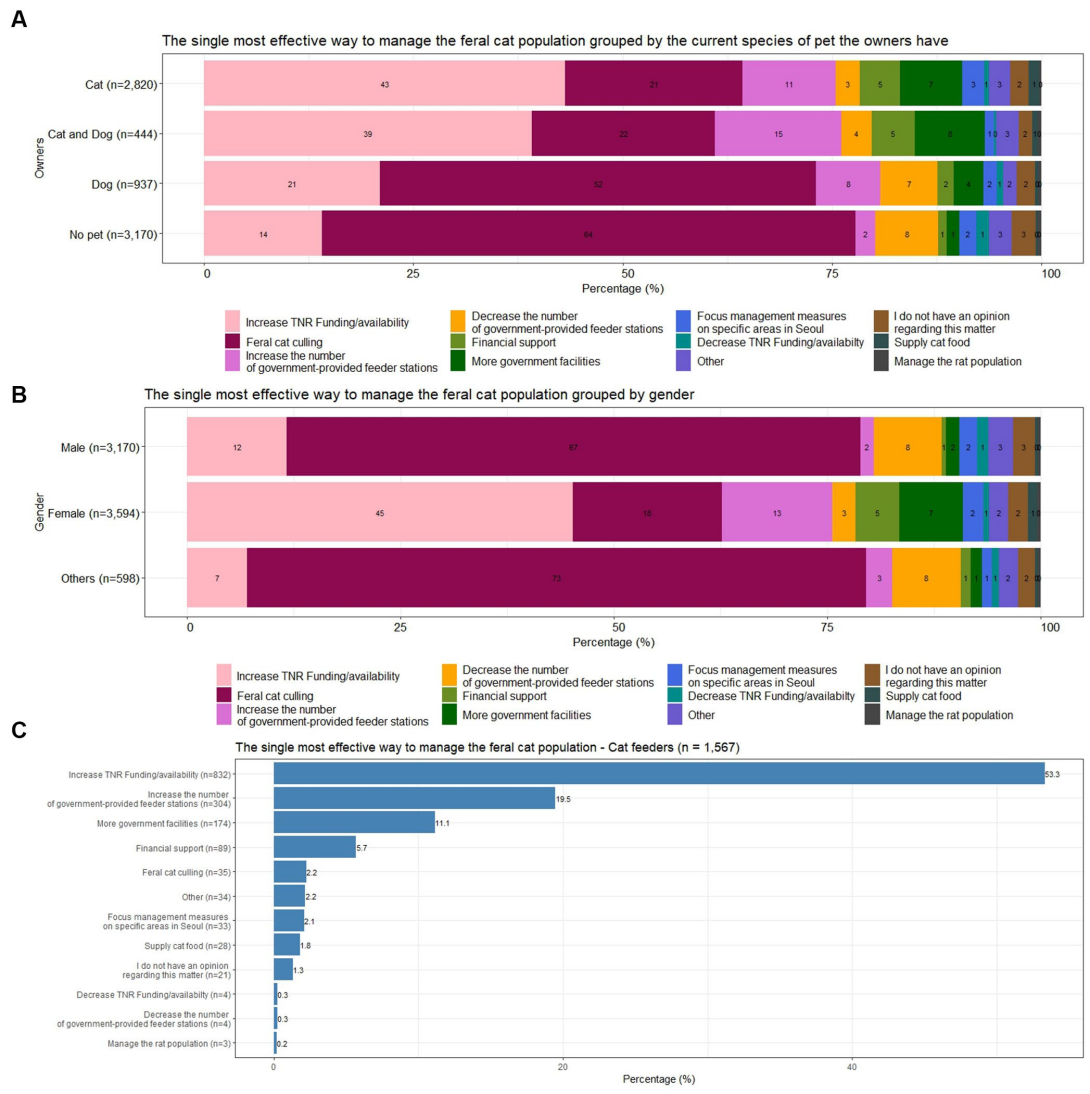
**(A)** The single most effective way to manage the feral cat population grouped by species of pet. **(B)** The single most effective way to manage the feral cat population grouped by gender. **(C)** The single most effective way to manage the feral cat population according to cat feeders.

Cat owners and cat and dog owners selected the single most effective way to manage the feral cat population as “Increase TNR funding/availability” (43, 39%), as shown in Figure 3A. However, for the same question, dog owners and people who did not have a pet selected “Feral cat culling” (52, 64%). When investigating the answers by gender group, shown in Figure 3B, males and the other gender group selected “Feral cat culling” as the most effective way as well (67, 73%). Male cat owners also selected “Feral cat culling” (261/567, 46%) as the most effective way to manage the feral cat population, whereas female cat owners answered “Increase TNR funding/availability” (1,070/2,095, 51%).

Cat feeders who answered that they had experienced government or private feeder stations for feral cats at least once, strongly favored “Increase TNR funding/availability” (832/1,567, 53%), shown in Figure 3C. They supported “Increase the number of government-provided feeder stations” (304/1,567,19.5%) and “More government facilities” (174/1,567, 11.1%) relatively more often than other pet owners.

**Figure 4:**
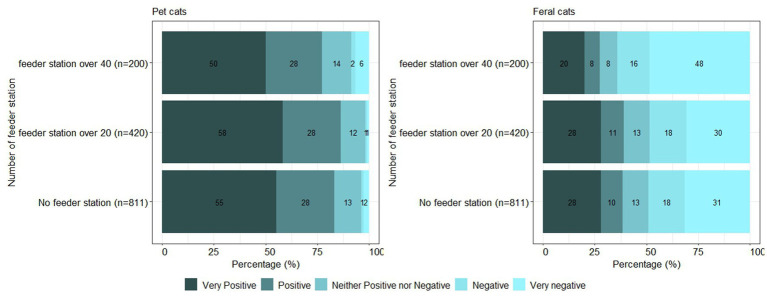
Attitudes toward pet cats and feral cats depending on the number of public feeder stations (*n* = 1,431).

According to the information provided by the Animal and Plant Quarantine Agency in Korea, at least 40 feral cat feeder stations were installed in 2 of 25 districts (Gangdong-gu and Gangnam-gu) in Seoul. Seven other districts (Dongdaemun-gu, Gwanak-gu, Jongno-gu, Jung-gu, Mapo-gu, Seocho-gu, and Seodaemun-gu) had 20–29 feral cat feeder stations. The other 16 districts had no feeder stations currently operating. After recategorizing the districts as three groups (no feeder station, feeder stations ≥20–39, feeder stations ≥40), participants’ attitudes toward pet cats and feral cats are shown in Figure 4, for the three categories of districts. Participants from the districts with at least 40 feeder stations had the highest percentage (48%) of answers as “very negative” toward feral cats. Current attitudes toward pet cats and feral cats showed significant differences among the three district groups (pet cats: *p* = 0.015; feral cats: *p* < 0.001). However, with pair-wise comparisons, the participants’ answers between districts with no feeder stations and districts with at least 20 feeding stations showed no significant differences in attitudes toward both pet cats (*p* = 0.4) and feral cats (*p* = 0.28).

For the open-ended question inquiring whether participants had experiences adopting cats in animal shelters in Korea, 74% (440/595) of the participants who answered “yes” to that question wrote their opinions in words. Most of the answers were about when and from which animal shelters they adopted the cats. They often mentioned the cats they adopted were babies so they could not leave them in the shelters. In addition, many of them said they decided to adopt the cats after hearing that the abandoned cats in the animal shelters would be euthanized after the certain amount of time if not adopted by anyone.

## Discussion

In this research, people who had at least one cat reported more positive attitudes toward feral cats than people who had no cats. The attitudes toward feral cats of people with only dogs were intermediate between people with cats and people with not pets, more resembling those of people without pets; attitudes of people with only dogs were less positive to cats than our hypothesis had predicted. A larger proportion of females than males kept cats, and the females were extremely more supportive of TNR and opposed to culling and other less humane methods than the males. In addition, there was a correlation between the number of city-provided feeding stations and people’s attitudes toward feral cats, but only in the areas with at least 40 feeder stations. Contrary to our hypothesis, people living in areas with many city-provided feeding stations had very negative attitudes to feral cats. More investigation would be required to assess whether the extremely negative attitudes had preceded or been increasing since adding the feeding stations.

Many other studies have investigated attitudes toward animals, and among them, some early studies focused specifically on attitudes to feral cats. In rural and non-rural Victoria, Australia, 22% of randomly selected respondents engaged in some type of semi-ownership behaviors with cats, primarily feeding (28). A survey of randomly selected households in the southeastern U.S. found that cat sanctuaries were most highly endorsed (56%) as a method to reduce feral cat populations, while TNR was supported almost as much (49%), and capture with euthanasia also had some substantial support (44%: 33). Cat owners more often opposed cat licensing and impounding stray cats, supported TNR, and were less concerned about water pollution. Working in Tel Aviv, Israel, Finkler and Terkel focused on the behaviors of cat owners that may contribute to cat overpopulation; education, income, gender, and age all were significant: less educated, older women being more likely to feed stray cats (32). Persons who did not neuter their cats also let their female cats give birth and allowed their non-neutered cats to roam: also abandoning non-neutered cats more frequently. Most of these cat-owning respondents also fed stray cats, and most of these cat owners did not neuter the strays. These authors in Israel proposed several measures to improve the level of knowledge and awareness among cat owners regarding cat overpopulation issues. A California study estimated comparisons of feces produced by outdoor pet cats vs. feral cats, finding that owned cats were responsible for 72% of the outdoor fecal deposition (23). Cat owners, more often than non-owners, opposed cat licensing and impounding stray cats and supported TNR. Studies in Brooklyn, New York, and Rome, Italy suggest that semi-owners, feral cat feeders, who intentionally provide food or other benevolent actions for the health and fitness of cats, contribute toward the overpopulation of cats in urban environments (33, 34). A similar conclusion was put forward in a recent study in Tennessee, where authors concluded that the people feeding the cats needed to be persuaded to provide less food, otherwise some cats would need to be euthanized (35).

Some community efforts have had positive effects. People managing the colonies of feral cats studied in Rome, Italy, compared with 20 years earlier, had improved their methods to assure hygiene, including removal of rubbish and neutering of cats, resulting in stable feral cat colony sizes; this reflected a somewhat increased cooperation between cat lovers and the public veterinary services (34). Unified efforts within UK communities resulted in better cooperation, with residents assisting in locating unowned cats and making progress with neutering them; the program enhanced the confidence and self-esteem of participants (36). The Amsterdam Stray Cat Foundation furthers the concept of supporting stray cats and their humans; their view is that humans provide care for the cats and cats also take care of humans, providing significant responsibility for the volunteers involved (37). A cautionary note is that stray cats in Japan, cared for as community cats with high welfare standards, still had numerous health problems, including one-sixth of the cats being FIV-positive (38). Yet, it seems that urban communities need to address the reality of public opinion, which is overwhelmingly in favor of “no-kill” shelters (39).

The opinions on feral cats for people living in very obviously environmentally vulnerable environments, where the lives of wild animals are jeopardized, are likely to differ from those of people living in urban centers (40). Feeders of feral cats in an early study in Oahu, Hawaii, were generally pet owners– more specifically middle-aged women living with their spouses, who had been feeding the feral cats for 2 to 4 years and sought to get them neutered (41). Crawford argued that TNR is not ethical for the welfare of wildlife in Australia; rather, strategies such as targeted adoption, early-age desexing, community education initiatives, and responsible pet ownership have greater promise (42).

In our study, we focused on relationships between pet ownership and attitudes toward feral cats. A study of young pet owners aged 9–19 years in an earlier study in Chicago also reported higher empathy and more favorable attitudes toward cats than non-owners; these young pet owners also reported lower delinquency (43). The relationship between cat ownership and positive attitudes to feral cats was generally supported by our study, that is, cat owners were more positive toward feral cats than non-cat owners.

Some semi-owners in this study managed private cat feeding stations, and the city of Seoul also provides feeding stations to manage feral cats and reduce conflict among feral cats, people who dislike feral cats, and people who like feral cats. In this study, the number of city-provided feeding stations in districts and the attitudes of people living in the districts toward feral cats were compared. In districts with more than 40 feeding stations, people showed more negative attitudes toward feral cats. As mentioned, It is unclear whether the extremely negative views preceded or followed the introduction of so many feeding stations. Gaining consensus on the management of outdoor cats also was found to be difficult in a study on a Japanese island (44). Increasing numbers of feral cats perhaps are likely in the future as more people get pet cats; in the United States, an early study concluded that there were almost as many feral cats as pet cats (45). But in contrast, a recent estimate of the number of unowned cats in the United Kingdom was almost 250,000, whereas pet cats were thought to be more than two million (46). A more substantial effort to estimate the number of domestic cats in an urban area was conducted by the Washington DC Cat Count: a collaboration of animal welfare organizations and wildlife scientists with extensive methodologies (47). Analyses of these extensive data showed that only 3% of the cats were feral, living outside fulltime, and the total number of cats was far higher than a previous estimate (48).

In this study, the four categories of people (cat only, cat and dog, dog only, and no pet) reported different opinions about the single most effective way to manage the feral cat population. Those who had at least one cat favored increasing TNR funding/availability, but those who did not have a cat most often preferred culling as the solution. A recent study of over 4,000 respondents, in Flanders, Belgium, differed from the results here in finding no effect of cat ownership on these opinions but instead, found that the attitudes toward cats, residence, and gender affected their preferences for managing stray cats (27). A strong majority of these Belgian respondents supported responsible household cat ownership and converting stray cats to “community cats”; these preferences were given especially by females, cat-lovers, and families without children. Killing stray cats and taking no action were least supported. These recent results are consistent with earlier research showing that caregivers have a strong bond with their feral cats (49). As also found in a study in Georgia where most people preferred sanctuaries over TNR, people’s attitudes are more important than experiences or knowledge for their ideas about managing stray cats (50).

### Limitations

This study has some limitations in terms of recruiting the survey participants and analyzing the data related to classifying the districts by the number of feeding stations. Since the present study recruited the participants mainly thorough social media outlets, participation bias may be present. Cat owners that had easy access to the social media would be more likely to answer the questions so the answers may not reflect the views of all pet and non-owners in Seoul. In addition, classifying the districts by the density of pet or feral cats would be more appropriate to reflect the attitudes on pet and feral cats. However, Korea only started to recommend that cat owners register their pet cats in 2018. Although registration of dogs is required, registering cats currently is elective, so obtaining the data regarding the density of cats in districts was not possible.

## Conclusion

Our study may contribute to understanding the relationships between feral cats and humans and resolving conflicts in the future. The results reveal the complexity of factors influencing people’s attitudes to pet and feral cats, with pronounced differences associated with pet ownership status, sex of the respondent, and characteristics of the neighborhood. Despite the recency of extensive petkeeping in South Korea and the density of housing in Seoul, these results generally are consistent with findings in other parts of the world where petkeeping is a longstanding practice. Females who own cats are most sympathetic to feral cats and could be prospects for participating in TNR programs. Further prospective studies could reveal details on when feeding stations are beneficial in neighborhoods and when they may increase problems with feral cats.

## Data availability statement

The original contributions presented in the study are publicly available. This data can be found here: https://figshare.com/articles/dataset/_strong_strong_Attitudes_and_practices_toward_feral_cats_of_male_and_female_dog_or_cat_owners_and_non-owners_in_Seoul_South_Korea/23646750/1.

## Author contributions

CK and LH initially conceived and designed the study, with bibliographic assistance from JL. CK collected the data. S-AK and SC compiled and analyzed the data. SC performed the statistical analyses provided ongoing edits for all manuscript drafts. JL surveyed relevant Korean resources. S-AK and LH drafted the initial manuscript. All authors then edited the manuscript. All authors contributed to the article and approved the submitted version.
